# To teach or not to teach? Assessing medical school faculty motivation to teach in the era of curriculum reform

**DOI:** 10.1186/s12909-022-03416-5

**Published:** 2022-05-12

**Authors:** Elizabeth R. Hanson, Eric A. Gantwerker, Deborah A. Chang, Ameet S. Nagpal

**Affiliations:** 1 Department of Pediatrics Joe R. and Teresa Lozano Long School of Medicine, UT Health San Antonio, San Antonio, TX USA; 2 Department of Otolaryngology - Head and Neck Surgery, Donald and Barbara Zucker School of Medicine at Hofstra/Northwell, New Hyde Park, New York, NY USA; 3 Office for Undergraduate Medical Education, Joe R. and Teresa Lozano Long School of Medicine, UT Health San Antonio, San Antonio, TX USA; 4 Department of Orthopaedics & Physical Medicine, Medical University of South Carolina, Charleston, SC USA

**Keywords:** Medical education, Faculty development, Curriculum, Pre-clinical, Teaching motivation, Self-determination theory

## Abstract

**Background:**

Medical schools have undergone a period of continual curricular change in recent years, particularly with regard to pre-clinical education. While these changes have many benefits for students, the impact on faculty is less clear.

**Methods:**

In this study, faculty motivation to teach in the pre-clinical medical curriculum was examined using self-determination theory (SDT) as a framework. Basic science and clinical faculty were surveyed on factors impacting their motivation to teach using validated scales of motivation as well as open-ended questions which were coded using self-determination theory (SDT) as a guiding framework.

**Results:**

Faculty reported that teaching activities often meet their basic psychological needs of competence, autonomy, and relatedness. Professors were more likely than associate professors to report that teaching met their need for autonomy. Faculty were more motivated by intrinsic as compared to external factors, although basic science faculty were more likely than clinical faculty to be motivated by external factors. Motivating and de-motivating factors fell into the themes *Resources, Recognition and Rewards, Student Factors, Self-Efficacy, Curriculum, Contribution,* and *Enjoyment*. The majority of factors tied to the faculty’s need for relatedness. Based on these findings, a conceptual model for understanding medical school faculty motivation to teach was developed.

**Conclusions:**

Assessing faculty motivation to teach provided valuable insights into how faculty relate to their teaching roles and what factors influence them to continue in those roles. This information may be useful in guiding future faculty development and research efforts.

**Supplementary Information:**

The online version contains supplementary material available at 10.1186/s12909-022-03416-5.

## Introduction

The last 20 years have marked a period of major curricular change in medical education with increased integration of clinical and basic science content, incorporation of active learning techniques, and a focus on interdisciplinary and team-based education [1, 2]. The pace of this change has not slowed in recent years; as of 2018, 84% of U.S. medical schools were either planning, undertaking, or had recently undergone a major curriculum reform. The majority of these reforms involved the pre-clinical years and often included increased content integration and increased use of educational technology [3].

These changes, designed to meet student needs, also have profound implications for faculty. Reforms often result in a shift in the role of the teacher and what is considered good teaching, leaving many faculty feeling challenged and uncertain [4]. The focus on a student-driven, problem-based approach often results in fewer opportunities for faculty to showcase their own expertise [5]. The drive for more centralized control of curricula and less faculty-student contact can result in conflicts between teaching faculty and curriculum leaders. While faculty recognize gains with a centralized structure, they experience losses in decreased autonomy and personal engagement with students [6]. Thus, curriculum reforms risk unintentional decreased faculty motivation and engagement. Educators from specific disciplines that previously ran distinct courses may perceive these changes as placing decreased importance on their subjects and expertise; although the ultimate impact may be to increase appreciation for the clinical relevance of their fields through vertical integration [7].

To best support the success of curricular reform, it is critical to examine the factors that influence medical school faculty motivations to teach. This is particularly important because teaching is often not subject to extrinsic financial rewards as compared to other academic or clinical pursuits; in fact, focus on teaching may lead to a delayed promotion for academic faculty [8, 9]. Understanding what motivates faculty to teach in the absence of extrinsic rewards is critical to maintaining and fostering a strong teaching workforce. Additionally, faculty motivation has been positively associated with engagement in faculty development activities and utilization of teaching best practices. Given the complexity of integrated curricula, these practices are key to successful curriculum reform [10, 11].

Self-determination theory (SDT) offers a useful framework to contextualize motivation for medical school teaching faculty as it encompasses the theories of human motivation of individuals to engage with certain behaviors. SDT states that “human beings have a natural tendency to develop autonomous regulation of behaviour and are intrinsically motivated to learn and to take on challenges” [14, pg 961]. SDT bases motivations on three basic psychological needs that necessitate being satisfied, namely, *autonomy*, *competence*, and *relatedness*. *Autonomy* is the ability to make choices for oneself. *Relatedness* is the need to feel a part of some greater societal group and create close relationship bonds. *Competence* refers to the need to feel capable and successful in facing challenges (of skills, knowledge, etc.) [12–14]. Organismic Integration Theory (OIT), a subset of SDT, discusses motivational factors that exist along a spectrum from amotivation (no motivation) to extrinsic motivation (external rewards) to intrinsic motivation (inherent interest in an activity). Extrinsic motivation stems from incentivizing individuals to engage in activities for external rewards or avoidance of punishment (monetary, recognition, etc.). Intrinsic motivation is the desire to engage in an activity due to the inherent fulfillment of the activity itself. Intrinsic motivation is the ideal state and is characterized by terms like curiosity and enjoyment and is fostered by satisfying the basic psychological needs of *autonomy*, *competence*, and *relatedness*. Importantly, people in this intrinsically motivated state will engage in activities even without external rewards. Alternatively, circumstances that undermine *competence*, *autonomy*, or *relatedness* threaten to undermine intrinsic motivation, leading either to amotivation or excessive reliance on external rewards to drive people toward the activity [13, 14].

There is a growing body of work utilizing SDT to understand what motivates academic faculty to engage in scholarly work, including both research and teaching [15–19]. One recent large study applied the principles of SDT to examine motivation to teach for academic faculty across institution types. However, this study included a small proportion of health professions faculty (8.7%), most of whom were non-physicians [11]. For medical school faculty, SDT may be particularly important to consider when student-centered curricular reforms may fail to consider the basic needs of the faculty, particularly with the competing demands of research and clinical work [5]. Many studies of medical school faculty that addressed motivation to teach have focused exclusively on the clinical teaching setting. In these studies, examples of motivating factors included the responsibility to help students become good doctors, enjoying the challenges of teaching, and inspiration from mentors, whereas, de-motivating factors included lack of involvement in course design, competing demands, lack of recognition/compensation, and lack of gratitude or respect from students [20, 21].

The pre-clinical phase of medical education refers to the initial portion of a medical school curriculum that is taught primarily in classroom and laboratory settings and covers core concepts in basic science and clinical medicine. In US medical schools, the pre-clinical phase is typically the first 18–24 months of the medical school curriculum. To our knowledge, there have not been any studies examining the motivation of basic science and clinical faculty during the pre-clinical phase of medical education. Research in this group is timely given the high proportion of educational reforms that specifically target the pre-clinical years [3]. In addition, pre-clinical faculty are typically a mix of basic scientists, physician-scientists, and clinician-educators, which may offer unique perspectives and challenges when it comes to motivating factors for teaching. In this initial study, faculty at one large institution were surveyed to explore the sources of motivation to teach in the pre-clinical curriculum and the specific factors that promote or undermine that motivation.

## Materials and methods

### Sample

Faculty who taught in the pre-clinical curriculum within the last 5 years at the UT Health San Antonio Joe R. and Teresa Lozano Long School of Medicine were surveyed. The 5-year mark was selected because that marked the first year of the current curriculum. This 20-month pre-clinical curriculum is comprised of three foundational modules (which cover the basic sciences and anatomy) followed by eight organ system modules. Running concurrently are two longitudinal modules, Clinical Skills and Medicine, Behavior and Society (MBS). All modules excluding Clinical Skills are taught predominantly in the classroom setting via a combination of lecture, lab, and team-based learning strategies. Basic science and clinical faculty from the foundational modules, organ system modules, and the MBS module were included in this study. Faculty who taught exclusively in the Clinical Skills module were excluded as the teaching strategies of that course differ significantly from the other modules and more closely mirror the clinical learning environment. Faculty were also excluded if they no longer had any active affiliation with the institution. This study was granted exempt status by the UT Health San Antonio IRB.

### Survey

The survey consisted of 31 items, including five demographic questions, two previously published 12-item scales assessing basic psychological needs and motivation respectively, and two open-ended questions for the qualitative component. The two 12-item scales were used with author permission exactly as published by Stupinsky et al. in 2017 [22], who created the scales by adapting them from the Work-related Basic Needs Satisfaction Scale (W-BNS) [23] and a scale of self-determination in the workplace [24]. Both of the adapted scales were subsequently validated in a large multidisciplinary sample of university faculty [11]. The two open-ended questions were worded as follows: 1) What are the most important factor(s) that would motivate you to take on a new teaching role at the medical school? 2) What factors are most likely to steer you away from taking on a new teaching role? The survey was administered via email using the Qualtrics system (Provo, UT).

### Quantitative analysis

Data were analyzed using IBM SPSS Statistics 26 software (Armonk, NY) to compare faculty scores on basic psychological needs and motivation. Independent-samples t-tests were conducted to compare mean scores by faculty type (clinical and basic science), teaching time (taught within the past 12 months or not), and gender identity (males and females; other not included in this analysis due to low n). One-way between-groups analysis of variance (ANOVA) were used to explore the both the impact of faculty rank and tenure status on basic psychological needs and motivation.

### Qualitative analysis

Authors EG and DC independently analyzed narrative comments from the two open-ended survey questions to identify motivational factors (codes). The codes were grouped into themes by examining the quotes that fell under each code and how they related to one another. Organismic integration theory (OIT) [14], a sub-theory of SDT, was used as a guiding theory during the coding process, and each code was assigned a level of motivation according to the OIT framework. In keeping with the quantitative scale validated in the larger university faculty group [11], the coders did not attempt to distinguish the integrated level of motivation, and rather coded comments based on a four-level model: External (fully external: focus on rewards and avoidance of punishments), Introjected (somewhat external: focus on what is expected and approval), Identified (somewhat internal: focus on values and goals), and Intrinsic (fully internal: focus on enjoyment and interest).

EG and DC then reviewed each other’s codebook and identified areas of differing opinions until a consensus was reached. Motivating and demotivating factors resulted in a common coding scheme due to a high overlap of themes resulting from the two questions. The codebook and comments were then given to a third author (EH) for coding and review who only made minor suggestions to clarify certain themes and adjust several OIT designations. All authors then discussed these suggestions to consensus, resulting in the final coding data. The final coding data were then used to develop a conceptual model that visually integrated the themes within the OIT framework to explain the factors influencing faculty motivation to teach in the pre-clinical curriculum.

## Results

### Demographics

Faculty survey response rate was 43% (108 of 250) for the full survey with gender identified as 52% male, 46% female, and 2% unspecified/other. Of the respondent faculty, 90% indicated that they had taught medical students in the past 12 months. Faculty role was 77% and 23% for clinical and basic science respectively with ranks identified as 34% professor, 34% associate professor, 27% assistant professor, 5% instructor; 33% tenured, 4% tenure track not yet tenured, 67% non-tenure track.

### Basic psychological needs

On average faculty reported that teaching was “often = 3” to “very often = 4” meeting their basic psychological needs of *competence* (Mean 3.49, SD 0.49), *autonomy* (Mean 3.26, SD 0.52), and *relatedness* (Mean 3.20, SD 0.66). In the faculty rank analysis, professors reported a significantly higher perception that teaching was meeting their need for *autonomy* when compared to associate professors (3.38 vs. 3.05, *p* = 0.004). The Cohen’s d (d = 0.6) indicated a medium effect size. No other statistically significant differences were detected between groups at the *p* < 0.05 level for the average scores for *competence*, *autonomy*, or *relatedness* (Table 1).

**Table 1:** Frequency^a^ with which teaching meets faculty basic psychological needs including competence, autonomy, and relatedness

	Category (n)	Competence Mean (SD)	Autonomy Mean (SD)	Relatedness Mean (SD)
Overall	All Faculty (107)	3.49 (0.49)	3.26 (0.52)	3.20 (0.66)
Faculty Rank	Professor (38)	3.59 (0.41)	3.41 (0.43)*	3.39 (0.54)
	Associate Professor (35)	3.46 (0.52)	3.07 (0.55)*	2.99 (0.66)
	Assistant Professor (29)	3.41 (0.54)	3.44 (0.51)	3.26 (0.76)
	Instructor (4)	3.40 (0.46)	2.95 (0.42)	3.27 (0.54)
Faculty Type	Basic Science (23)	3.61 (0.46)	3.17 (0.54)	3.17 (0.67)
	Clinical (83)	3.45 (0.50)	3.31 (0.51)	3.23 (0.65)
Gender Identity	Female (50)	3.45 (0.52)	3.24 (0.58)	3.34)0.69)
	Male (55)	3.54 (0.46)	3.35 (0.44)	3.15 (0.58)
Most Recent Teaching	< 12 months (98)	3.50 (0.49)	3.29 (0.52)	3.23 (0.67)
	> 12 months (9)	3.32 (0.48)	3.21 (053)	3.16 (0.47)

^a^Scale for survey items **4** *Very often* **3** *Often* **2** *Sometimes* **1** *Never*

^*^p = 0.004

### Motivation

Faculty more often cited intrinsic factors (M = 3.65, SD = 0.4) over external factors (M = 1.94, SD = 0.68) as motivators for teaching. On the faculty type analysis, basic science faculty were more likely to indicate external factors as motivators for teaching (2.40 vs 1.80, *P* < 0.001). No other statistically significant differences were detected between groups at the *p* < 0.05 level for the average scores for intrinsic, identified, introjected, or external factors (Table 2).

**Table 2:** Extent^a^ to which factors along OIT spectrum motivate faculty to teach ranging from intrinsic to identified to introjected to external motivations

	Category (n)	Intrinsic Mean (SD)	Identified Mean (SD)	Introjected Mean (SD)	External Mean (SD)
Overall	All Faculty (107)	3.65 (0.43)	3.55 (0.51)	1.92 (0.84)	1.94 (0.68)
Faculty Rank	Full Professor (38)	3.69 (0.44)	3.56 (0.52)	2.13 (0.95)	2.01 (0.59)
	Assoc Professor (35)	3.59 (0.44)	3.58 (0.47)	1.68 (0.73)	1.86 (0.71)
	Assist Professor (29)	3.71 (0.41)	3.60 (0.48)	2.00 (0.76)	1.97 (0.69)
	Instructor (4)	3.75 (0.33)	3.10 (1.02)	1.00 (0.00)	1.38 (0.75)
Faculty Type	Basic Science (23)	3.73 (0.41)	3.61 (0.37)	1.74 (0.76)	2.40 (0.74)*
	Clinical (83)	3.64 (0.43)	3.53 (0.55)	1.95 (0.85)	1.80 (0.59)*
Gender Identity	Female (50)	3.67 (0.43)	3.59 (0.52)	1.79 (0.81)	1.85 (0.67)
	Male (55)	3.64 (0.44)	3.54 (0.51)	2.04 (0.87)	1.95 (0.61)
Most Recent Teaching	< 12 months (98)	3.66 (0.43)	3.57 (0.50)	1.90 (0.83)	1.92 (0.68)
	> 12 months (9)	3.60 (0.49)	3.40 (0.64)	2.12 (0.90)	1.92 (0.50)

^a^Scale for survey items: 4 *Very much* 3 *Quite a bit* 2 *Some* 1 *Very little*

^*^p < 0.001

### Qualitative analysis

Overall, respondents discussed several factors that either led them towards or away from pre-clinical teaching opportunities. These fell into seven themes: *Resources, Recognition and Rewards, Student Factors, Self-Efficacy, Curriculum, Contribution,* and *Enjoyment*. The first five themes were found in both the motivating and demotivating factors; whereas the themes of *Contribution* and *Enjoyment* were found exclusively in the responses to motivating factors (Table 3).

**Table 3:** Qualitative coding scheme of factors impacting faculty motivation to teach and the mapping to OIT level and SDT needs of autonomy, relatedness, and competence

Theme	Codes (M = Motivating, D = Demotivating)	OIT Level	SDT Basic Need
Resources	Clinical and Administrative Responsibility (M)	External	Autonomy
	Admin support (M)	External	Relatedness
	Collegial Support (M)	Introjected	Relatedness
	Fiscal Responsibility (M)	Identified	Autonomy
	Expectations for Revenue (D); Lack of Protected Time (D)	External	Autonomy
	Scheduling (D)	External	Relatedness
Recognition and Rewards	Personal Compensation (M)	External	Relatedness
	Recognition of Value to Institution (M); Student Recognition (M)	Introjected	Relatedness
	Personal Development (M)	Identified	Competence
	Lack of Compensation (D)	External	Relatedness
	Lack of Recognition (D); Lack of Institutional Value (D)	Introjected	Relatedness
Student Factors	Student Engagement (M); Lack of Student Effort (D); Lack of Student Respect (D)	External	Relatedness
Self-Efficacy	Expertise Validation -Individual (M)	Introjected	Relatedness
	Content Expertise (M)	Introjected	Autonomy/ Competence
	Lack of Perceived Expertise (D)	Introjected	Competence
Curriculum	Curriculum Awareness (M)	Introjected	Autonomy
	Content Interest (M)	Introjected	Autonomy/ Competence
	Instructional/ Content Autonomy (M); Instructional Methodology Autonomy (M)	Identified	Autonomy
	Curriculum Philosophy (D); Teaching Style Autonomy (D); Content Autonomy (D)	Introjected	Autonomy
Contribution	Sense of Duty (M)	Introjected	Relatedness
	Sense of Impact (M); Share Expertise (M)	Identified	Relatedness
Enjoyment	Sense of Fulfillment (M); Peer Collaboration (M);Love of Teaching (M)	Intrinsic	Relatedness

**Fig. 1:**
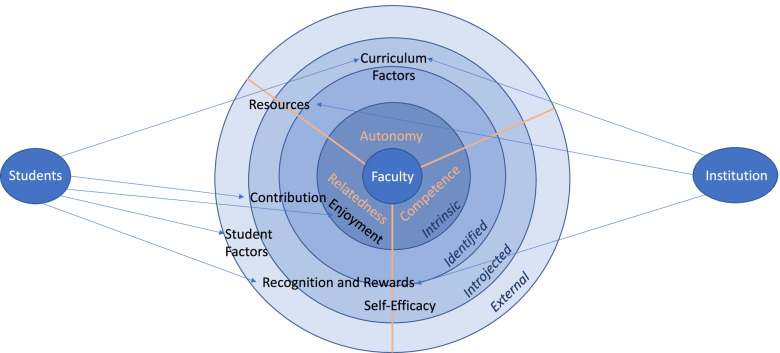
Conceptual Model of Factors Impacting Faculty Motivation to Teach in the Pre-Clinical Curriculum. Legend: Fig. 1 depicts factors motivating faculty to teach in the medical curriculum mapped according to type of motivation as well as the basic psychological need they address. As is evident, the majority of motivating factors tie to the faculty’s need for relatedness to both students and the teaching institution

For some themes, the codes spanned a portion of the OIT spectrum, such as the codes within the theme *Recognition and Rewards* which ranged from external to identified. For other themes, the codes all fell at the same OIT level, such as the theme *Enjoyment* which was entirely intrinsic. Furthermore, the codes clustered in each theme generally corresponded to one or two of the basic psychological needs. The OIT range and predominant basic psychological needs for each theme are noted in Table 2 and illustrated in the conceptual model (Fig. 1).

### Themes

#### Resources

Several respondents discussed resources at the departmental and institutional level noting that teaching can result in research and/or clinical productivity losses. Other comments focused on the individual resources needed to support teaching, such as administrative support, or time protected from other responsibilities. Codes in this theme mapped both to the SDT need for autonomy and relatedness.

- Motivating: “salary support to my division or department and administrative support to help with preparing for the teaching session.”
- Demotivating: “lack of time to prepare the lectures… expectation to hit target RVUs and how that impacts the time available to prepare”

#### Recognition and rewards

Although many faculty referenced a desire for financial rewards in the form of salary support for teaching, others focused on a desire for personal recognition and awards and some mentioned the intrinsic rewards from personal growth/experience. Several faculty from both basic science and clinical departments commented that teaching is not valued by the institution based on the lack of recognition for the importance of teaching and the disproportionate considerations for clinical revenue and grants when it comes to promotion/tenure. Codes in this theme were predominantly mapped to the SDT need for *relatedness*.

- Motivating: “additional pay (to my personal paycheck) for my time spent in the classroom AND time spent preparing lectures”; “teaching awards or opportunities for such, specifically coming from…students”; “experience[s] [that] combines value to the learner and/or program while also providing me a chance to learn and grow.”
- Demotivating: “The emphasis is on clinical production and those that teach are expected to do so in their 'spare' time.”; “the fact that my salary is based solely on the percentage of salary recovery from research grants, and not on teaching quantity or quality.”

#### Student-factors

Several faculty commented on the role that their interactions with students play in their motivation to teach. While student interactions are intertwined with other themes as well, factors specifically related to the students were engagement, effort, and respectfulness toward faculty. These again map closely to the SDT need for *relatedness*.

- Motivating: “being able to reach out to students and get them excited and engaged in learning and let them know this is a privilege”
- Demotivating: “awful comments I hear about students saying about faculty teaching”

#### Self-Efficacy

Another common theme was the presence or absence of self-efficacy (SE) as it relates to the teaching content or the educational techniques. Codes in this area mapped to the SDT need for *competence*, but also to *autonomy* and *relatedness*. This was the only theme that mapped to all three of the basic psychological needs.

- Motivating: “subject matter being something I am interested in and feel proficient in enough to teach”
- Demotivating: “lack of confidence in my ability to teach certain topics outside of my area of interest/expertise”; “an unfamiliarity with the perceived techniques”

#### Curriculum

A major theme was a desire for involvement in the process of changing the curriculum. Both content and instructional methodology were mentioned. Codes in this theme predominantly mapped to the SDT need for *autonomy*.

- Motivating: “more complete understanding of what the medical students are getting taught”; “freedom to focus on my areas of expertise, topics I think are valuable to fledgling physicians”
- Demotivating: “the administrative hassle… I did not like the way the new curriculum was set up and how I was supposed to find the time to interface with multiple other faculty members to just teach a subject.”

#### Contribution

Many faculty mentioned a desire to make an impact, share their expertise, or even expressing a feeling of a sense of duty to impart their knowledge and experience to the next generation of doctors. Codes in this theme were all under the motivating theme and predominantly mapped to the SDT need for *relatedness*.

- “I'd feel that I was making a contribution to medical student education from the basic science perspective. “
- “the ability to positively impact students that we are teaching to empower them to learn more and to become great medical providers.”
- “I consider it important to the profession to teach…”

#### Enjoyment

Some faculty expressed genuine enjoyment in teaching both in working with the students and collaborating with faculty. Codes in this theme were all also under the motivating theme and predominantly mapped to the SDT need for *relatedness*.

- “[The] ability to collaborate with colleagues is always fun for me.”
- “It is always a pleasure to work with students and see them blossom into competent physicians.”

#### Conceptual model

To synthesize the interplay of the factors impacting faculty motivation, we developed a conceptual model by overlaying the themes onto the basic psychological needs and OIT levels of motivation of the codes within those themes. We further tied the themes to the two stakeholder groups that were mentioned most frequently in the quotes as impacting faculty motivation: the students and the academic institution (Fig. 1).

## Discussion

This study assessed faculty motivation to teach in a pre-clinical curriculum at one large medical school using the SDT and OIT frameworks. The quantitative analysis demonstrated that faculty tended to be motivated by factors categorized as intrinsic or identified and that teaching met their basic psychological needs. This aligns with the comments from faculty who felt that they were contributing to the education of the students and had a pure enjoyment of teaching. This was enabled by their ability to have a sense of impact and create close relationships with the students. Based on SDT, we would predict that faculty who fall into these areas are more likely to continue to teach and have less reliance on external rewards. These findings are similar to the results of a recent study examining academic faculty motivation to teach across a variety of disciplines. In that study, faculty motivation was categorized as autonomous (a combination of intrinsic and identified) in 52% of faculty, introjected in 15% of faculty, and extrinsic in 33% of the faculty. Faculty who reported that their basic psychological needs were being met were more likely to fall in the autonomous motivation group [11].

Differences were found based on faculty demographics on two measures. Basic scientists were more likely than clinical faculty to be motivated by external factors. This may be related to the different ways in which faculty positions for basic science and clinical faculty are funded or could reflect unique environmental factors specific to the cultures of the two groups. In many institutions, basic science faculty are expected or assigned to teach at the medical or graduate school level, whereas clinical faculty may only engage in clinical teaching unless they specifically apply or volunteer to teach in the classroom setting. As such, clinical faculty engated in pre-clinical/classroom teaching may represent a subset that is already intrinsically motivated to engage in teaching at this level. In our study, professors were more likely than associate professors to indicate that teaching met their need for *autonomy*. This sense may stem from the likelihood of high-ranking faculty having an influence on the curriculum and/or faculty at this stage of their career being free to accept or reject teaching assignments without impact on promotion. Exploring these differences could be useful to better understand how faculty motivation varies by faculty demographics, which in turn could inform faculty development efforts.

The qualitative analysis demonstrated that although faculty value teaching, they also face external sources of frustration such as lack of funding, recognition, and resources to support teaching activities. They found it difficult to balance teaching activities with revenue-generating activities and perceived expectations from their department or institution. These frustrations place extrinsic motivations from other activities in direct conflict with those of teaching which may be even more intrinsic or altruistic. These are real-world implications that may stem from the challenges of properly quantifying the value of teaching to the institution and alotting the appropriate amount of financial support as has been previously explored in academic health centers [25]. Some faculty cited a perceived lack of effort and recognition by the students. Faculty often enter into a social contract with students and those that put in the effort to create educational experiences may not be reciprocated, which may undermine the social contract and relationship-building between faculty and students. Faculty also reported a lack of *competence* and/or *autonomy* with the curriculum change that had occurred, which required a pedagogical shift toward a more integrated curriculum incorporating active learning techniques. This is not surprising as faculty go through the identity shift from content deliverer to co-facilitator of learning in which they may not feel competent and feel a loss of control over their sessions. These themes were similar to those found in the qualitative studies of teachers in the clinical setting, although the clinical teachers had more of a focus on the end product of students as future physicians [20, 21].

A conceptual model was created to better understand the relationship between faculty motivation to teach, students, and the institution (Fig. 1). Viewed this way, the majority of the motivating factors, whether student or institutionally connected, were at least partly categorized as addressing the need for *relatedness*. By contrast, *relatedness* was the basic need that was least frequently indicated as being met based on our quantitative data. It is plausible, therefore, that faculty motivation may be improved by an intervention that enhances the feeling of connectivity to students and the institution. This is particularly important given the national trend of decreased medical student attendance at live lectures [26] and the recent shift to virtual education during the COVID-19 pandemic. Attention to structures that maintain a sense of *relatedness* for faculty with their peers and with the students despite the move to online teaching could be vital to maintaining faculty motivation to teach in this challenging new environment.

The lack of *competence* as expressed by some faculty supports the need for faculty development in modern teaching techniques to shift from lecturer to facilitator of active learning, especially using technology. Historical techniques of being the “sage on stage” often do not lead to adequate student engagement. This in turn may contribute to students no longer wanting to attend live classes, finding more value in watching recorded lectures and integrating outside resources while studying on their own. This can further contribute to the lack of *relatedness* as the faculty rarely engage with the students if they do not attend live sessions. Some lessons can be learned from examining the strategies employed by distance and e-learning faculty to engage with students asynchronously. Previous work examining faculty motivation in these settings identified that a combination of intrinsic and extrinsic motivating factors are important to maintain faculty motivation to teach when face-to-face interactions are lacking [27].

The lack of *autonomy* may also stem from this curriculum reform creating more of these active learning experiences for the students. Faculty being told to completely re-create educational sessions that they have used for years can lead to frustration and disengagement. This again may speak to the role of faculty development and involvement of these faculty in the curriculum reform understanding the underlying principles that schools wish to inculcate in their modern teaching.

This study has several limitations. As a single-institution study, the results may not be representative of the larger community, as it has been shown that faculty attitudes toward and responses to curriculum change vary by cultural context [28, 29]. However, as a large academic facility with a diverse teaching faculty, it is likely that the themes expressed by the faculty may resonate with others and this is reflected in studies performed at other institutions. Because sampling was limited to only to faculty who have taught in the last five years, important factors that have kept some faculty out of teaching altogether may have been missed. As the current study was limited to information collected via survey, faculty perceptions on how their motivation to teach intersects with their decisions to accept or decline new teaching opportunities have not yet been fully explored. This limitation is slightly mitigated by the relatively large sample size with an adequate response rate (43%) and the inclusion of open-ended questions. Future directions include a more in-depth exploration of these themes with faculty via focus groups, and an expansion of this study at other medical schools. Such a multi-institutional study would provide a sufficient sample size to further explore the potential differences in motivation by faculty demographics with the ultimate goal of providing a scaffold upon which future faculty development efforts can be focused.

## Conclusions

This initial study provides an important first step in understanding teaching faculty motivations for teaching in pre-clinical curriculum at a large medical school using the self-determination theory (SDT) and organismic integration theory (OIT) frameworks. Faculty remain strongly motivated to contribute to the education of the next generation; however, there are clear external sources of frustration such as lack of funding, recognition, and resources to support these activities and difficulty in balancing teaching activities with revenue-generating activities. Despite intrinsic motivations, external factors may lead to undermining the basic psychological needs of *autonomy, relatedness*, and *competence.* Leaders of curriculum reform should engage faculty early in the process and foster the development of expertise in newer teaching techniques to support the transition from content deliverer to facilitator of active learning. Leadership should also foster relationships amongst faculty and students and faculty with their colleagues. Failure to do so may be disempowering and lead to frustration and demotivation. Absence of protected time and compensation for teaching undermined institutional relatedness via a perceived devaluing of faculty efforts. Recognition, such as teaching awards, may ameliorate some of these concerns, but creative solutions should also be sought to improve funding and time for faculty teaching at the medical school. Finally, faculty engagement in teaching activities needs to be taken into account in faculty recruitment and promotion, with incentives provided for outstanding performance.

## Supplementary Information

**Additional file 1:** **Supplement 1. **Survey Questions for “To teach or not to teach? Assessing medical schoolfaculty motivation to teach in the era of curriculum reform”

## Data Availability

The datasets generated during and/or analyzed during the current study are available from the corresponding author on reasonable request.

## Abbreviations

ANOVA: Analysis of variance
MBS: Medicine, Behavior and Society Module
OIT: Organismic Integration Theory
SDT: Self-determination Theory
