# Supplementary material for: To teach or not to teach? Assessing medical school faculty motivation to teach in the era of curriculum reform
Source: BMC Med Educ. 2022 May 12;22:363. doi: 10.1186/s12909-022-03416-5 (PMC9096333; doi:10.1186/s12909-022-03416-5)
Supplement: Supplementary file 1 — Additional file 1: Supplement 1. Survey Questions for “To teach or not to teach? Assessing medical schoolfaculty motivation to teach in the era of curriculum reform” [file 12909_2022_3416_MOESM1_ESM.docx]

**Supplement 1: Survey Questions for “To teach or not to teach? Assessing medical school faculty motivation to teach in the era of curriculum reform”**

Survey Questions

1. Do you consider yourself to be primarily a basic science or clinical faculty member?
2. Clinical faculty
3. Basic Science Faculty
4. In the last 12 months, have you taught in the medical school pre-clinical curriculum?
5. Yes
6. No
7. What is your current academic rank?
8. Professor
9. Associate Professor
10. Assistant Professor
11. Instructor
12. What is your current tenure status?
13. Tenured
14. Tenure track, not yet tenured
15. Not-tenure track
16. What is your gender identity?
17. Female
18. Male
19. Other
20. Prefer not to answer

****Questions 6-17 use the following prompt and response options:***

*Prompt: In your teaching*, how often do you feel the following?

*Response options:* **4** *Very often* **3** *Often* **2** *Sometimes* **1** *Never*

1. I have a sense of freedom to make my own choices.
2. I have confidence in my ability to do things well.
3. The people I care about (students, colleagues, etc.) also care about me.
4. My decisions reflect what I really want.
5. I am capable at what I do.
6. I am supported by the people whom I care about (students, colleagues, etc.)
7. My choices express who I really am as a teacher.
8. I can competently achieve my goals.
9. I am close with people who are important to me (students, colleagues, etc.)
10. I do what really interests me.
11. I can successfully complete difficult tasks.
12. I experience warm feelings with the people I spend time with (students, colleagues, etc.)

****Questions 18-29 use the following prompt and response options:***

*Prompt:* To what extent are the following reasons for why you teach?

*Response options:* **4** *Very much* **3** *Quite a bit* **2** *Some* **1** *Very little*

1. It is pleasant to teach.
2. It is important for me to teach.
3. If I don’t teach I will feel bad.
4. My work demands that I teach.
5. I find teaching interesting.
6. Teaching allows me to attain work objectives that I consider important.
7. I would feel guilty not teaching.
8. Because my university/college obliges me to teach.
9. I like teaching.
10. Teaching is important for the academic success of my students.
11. I do not want to feel bad if I do not teach.
12. Because I am paid to teach.

**Questions 30-31 are open response:**

1. What are the MOST important factor(s) that would motivate you to take on a new teaching role at the medical school?
2. What factors are most likely to steer you away from taking on a new teaching role?

****Questions 6-29 were used with permission from a previously published study on faculty teaching motivation:***

Stupnisky RH, BrckaLorenz A, Yuhas B, Guay F. Faculty members’ motivation for teaching and best practices: Testing a model based on self-determination theory across institution types. Contemp Educ Psychol. 2018;53(January):15–26.
